# Sectoral Changes in Neuroretinal Rim Pallor Across Refractive Error

**DOI:** 10.1016/j.xops.2025.100705

**Published:** 2025-01-07

**Authors:** Fabian Yii, Samuel Gibbon, Tom MacGillivray

**Affiliations:** 1 Robert O Curle Ophthalmology Suite, Institute for Regeneration and Repair, The University of Edinburgh, Edinburgh, UK; 2 Centre for Clinical Brain Scienes, The University of Edinburgh, Edinburgh, UK

**Keywords:** Myopia, Refractive error, Papillomacular bundle, Neuroretinal rim pallor, Optic disc pallor

## Abstract

**Purpose:**

To investigate the association between spherical equivalent refraction (SER) and pallor in different neuroretinal rim (NRR) sectors.

**Design:**

Population-based cross-sectional study.

**Participants:**

Normal eyes of 24 057 healthy participants aged 40 to 70 years from the UK Biobank.

**Methods:**

Pallor in different NRR sectors was quantitatively derived from color fundus photographs using automated software. We first examined the association between SER and pallor in each NRR sector—controlling for age, sex, ethnicity (White vs. non-White), intraocular pressure, and mean blood pressure. We then incorporated disc–fovea distance (the shortest distance from the center of the disc to the fovea) and temporal arterial/venous concavity (extent to which the temporal artery/vein curved inwardly toward the fovea) as additional independent variables, as these features have been suggested to reflect the degree of axonal stretching at the posterior pole.

**Main Outcome Measures:**

Pallor in the temporal, temporal inferior, nasal inferior, nasal, nasal superior, and temporal superior sectors of the NRR.

**Results:**

Moving from the temporal sector to the temporal superior sector, NRR pallor varied in an asymmetrical U-shaped pattern, with the least pallor observed nasally. White participants tended to have paler NRR, but the association between SER and pallor did not differ between ethnic groups (no interaction effect between SER and ethnicity). Decreasing SER was associated with increasing pallor in all 6 NRR sectors (all *P* < 0.001), but the temporal (ß: −0.009, 95% confidence interval: −0.011 to −0.008) and temporal inferior (ß: −0.008, 95% confidence interval: −0.009 to −0.007) sectors exhibited the steepest increase. The rate of increase diminished by half toward the more nasal/central sectors, and by another half in the nasal-most sector. Consistent with these changes, increasing disc–fovea distance and temporal arterial/venous concavity resulted in up to 4 times as much pallor temporally compared with nasally. These retinal changes accounted for approximately ≥50% of the effect of SER on NRR pallor.

**Conclusions:**

Decreasing SER increases NRR pallor approximately 4 times faster temporally than nasally. The association between SER and NRR pallor is primarily attributable to changes in disc–fovea distance and temporal arterial/venous concavity. These findings suggest that the papillomacular nerve fiber bundle, linked to the temporal NRR, is most susceptible to myopic stretching.

**Financial Disclosure(s):**

The author(s) have no proprietary or commercial interest in any materials discussed in this article.

Driven in part by the established association between myopia and glaucoma,^1^^,^^2^ the influence of refractive error on optic nerve head (ONH) parameters has long garnered interest.^3^^,^^4^ The geometrical properties of the ONH in myopic eyes, in particular, have been the subject of investigation in numerous studies,^4^, ^5^, ^6^, ^7^, ^8^ but no research has objectively and quantitatively examined the influence of refractive error on pallor within the optic disc (OD).

Investigating changes in OD pallor—or more precisely, neuroretinal rim (NRR) pallor—has important implications for understanding myopia-related nonglaucomatous optic neuropathy (NGON), a condition characterized by central or paracentral visual field defects that are not attributable to macular pathology but rather to damage of the retinal ganglion cell axons.^9^^,^^10^ Neuroretinal rim (NRR) pallor is used as a main diagnostic criterion to differentiate NGON from glaucomatous optic neuropathy because the latter typically does not cause pallor in the remaining NRR.^11^ An understanding of myopic changes in pallor across different NRR sectors may provide insights into the pathophysiology of NGON, as nerve fibers corresponding to different NRR sectors are believed to exhibit varying susceptibilities to mechanical damage from myopic stretching at the posterior pole. Specifically, the papillomacular bundle—linked to the temporal NRR—has been conjectured to be more vulnerable to damage than the arcuate bundle.^9^^,^^12^ If so, one may hypothesize sectoral differences in the rate of change in pallor with refractive error, with the temporal NRR exhibiting a more rapid increase in pallor as refractive error increases in the myopic direction.

Additionally, OD–fovea distance and the concavity (degree of convergence) of temporal retinal vessels, both of which increase with greater myopic refractive error or axial length,^13^, ^14^, ^15^ are thought to reflect the degree of axonal stretching at the posterior pole.^9^^,^^12^^,^^16^ These retinal features, therefore, may be directly associated with NRR pallor, accounting for the possible effect of refractive error on NRR pallor. The present study aimed to test these hypotheses by investigating the association of refractive error—and the aforementioned retinal features—with pallor in different NRR sectors.

## Methods

### Study Participants

Study participants were sourced from a major biomedical database, UK Biobank, which has Research Tissue Bank approval from the North West Multi-Center Research Ethics Committee (06/MRE08/65). Therefore, the present study was exempt from obtaining separate ethical clearance. A detailed description of the UK Biobank is available elsewhere.^17^

Briefly, around 68 000 community-dwelling residents aged 40 to 70 years from across the UK participated in an extensive range of standardized physical and ophthalmic assessments between 2006 and 2010. These included systolic and diastolic blood pressure measurements using the Omron HEM-7015IT digital blood pressure monitor (OMRON Healthcare), 45° macula-centered color fundus photography using Topcon 3D OCT-1000 Mark II (Topcon Corp), visual acuity evaluation on a digital logarithm of the minimum angle of resolution chart (Precision Vision, LaSalle); auto-refraction/keratometry using Tomey RC-5000 (Tomey); and measurements of Goldmann-correlated intraocular pressure (IOP) with Ocular Response Analyzer (Reichert Corp). Note that only phakic participants who had never undergone any refractive or cataract surgery were invited to take part in the ophthalmic assessment.

After excluding both eyes of participants with hypertension (35 082 eyes), diabetes (2506 eyes), and heart conditions (2159 eyes), all of which were identified based on linked health care data,^18^ 95 062 eyes of 47 944 participants remained. Participants with chorioretinal, scleral, or optic nerve disorders (including glaucoma) in ≥1 eye were similarly excluded, leaving 92 160 eyes of 46 464 participants. A total of 37 255 eyes with missing or poor visual acuity (logarithm of the minimum angle of resolution >0.00) were also excluded to minimize the influence of cataract on pallor measurements, after which 54 905 eyes of 34 284 participants remained. Auto-refraction/keratometry or IOP data were not available for 2198 eyes, so these were further removed. Finally, we excluded 18 757 eyes that failed image quality control (more details in Supplemental Material S1 and S2, available at www.ophthalmologyscience.org), leaving 33 950 eyes of 24 057 participants for subsequent analysis.

### NRR Pallor, Disc–Fovea Distance, and Temporal Vessel Concavity

**Figure 1:**
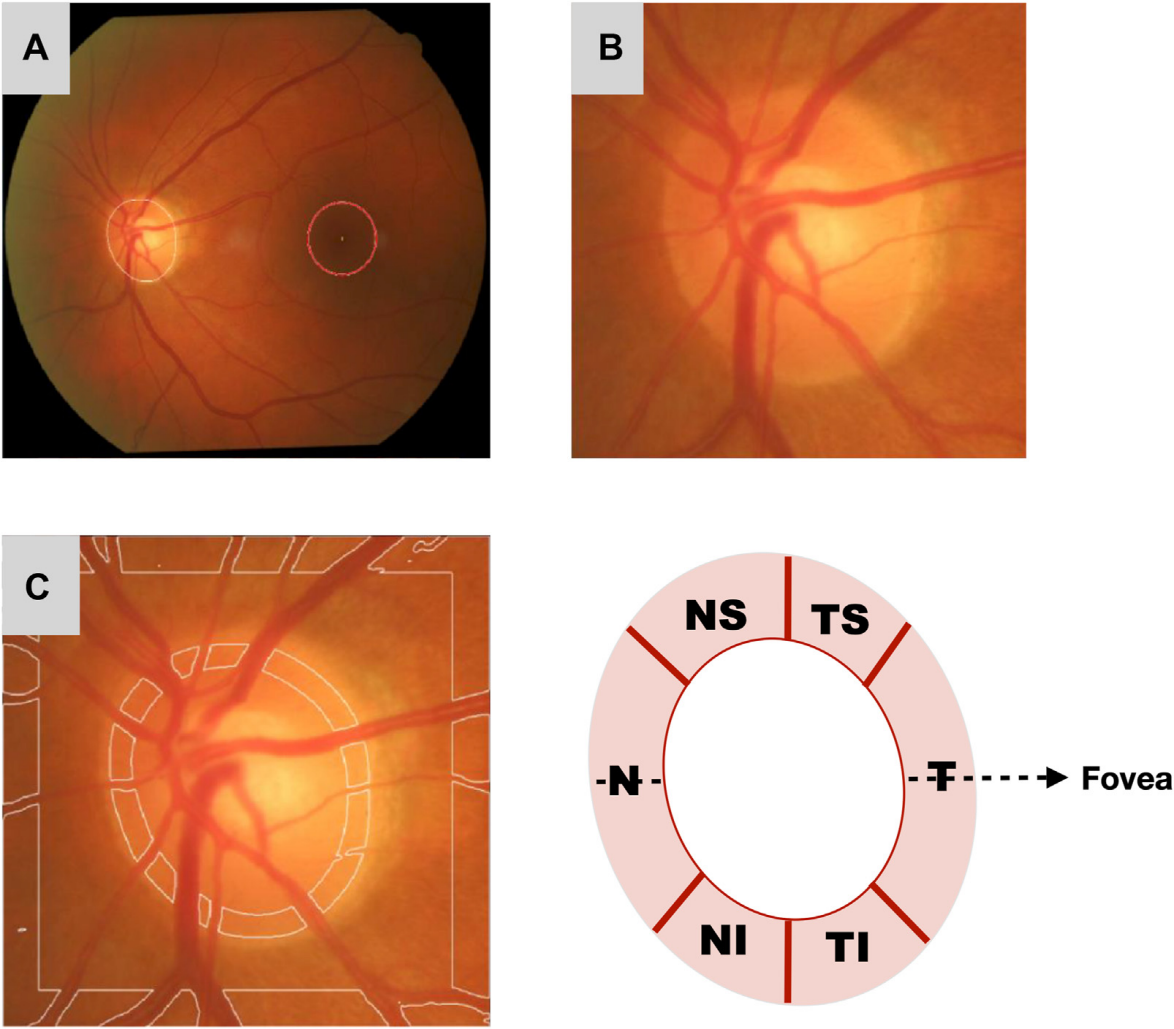
Brief overview of pallor measurements within the temporal (T), temporal inferior (TI), nasal inferior (NI), nasal (N), nasal superior (NS), and temporal superior (TS) neuroretinal rim sectors using a previously validated method.^19^**A,** The image is first rotated so that the optic disc and fovea lie along the horizontal axis of the image frame. **B,** The image is then cropped around the optic disc. **C,** Neuroretinal rim pallor is derived from the measurement region (excluding the retinal vasculature) delineated by the white lines within the optic nerve head (extending 30 pixels inward from the disc border), adjusted for background illumination in the control region (also excluding the retinal vasculature) located some distance away from the optic nerve head.

Neuroretinal rim pallor was quantitatively derived from color fundus photographs using fully automated software that we previously developed and validated,^19^ which has been applied to recent research.^20^ Briefly, the software segmented the OD, retinal vasculature, and fovea using deep learning before cropping the image around the OD. After this, pallor was computed by taking the ratio of the average intensity of nonvessel pixels within the NRR to that in the control region (Fig 1). A greater pallor value corresponded to a paler (less pinkish) NRR appearance. Neuroretinal rim pallor was computed both globally and separately in 6 different sectors—with the temporal sector extending 45° from either side of the OD–fovea axis, followed clockwise by the temporal inferior sector (45°–90°), nasal inferior (90°–135°), and so on (Fig 1).

**Figure 2:**
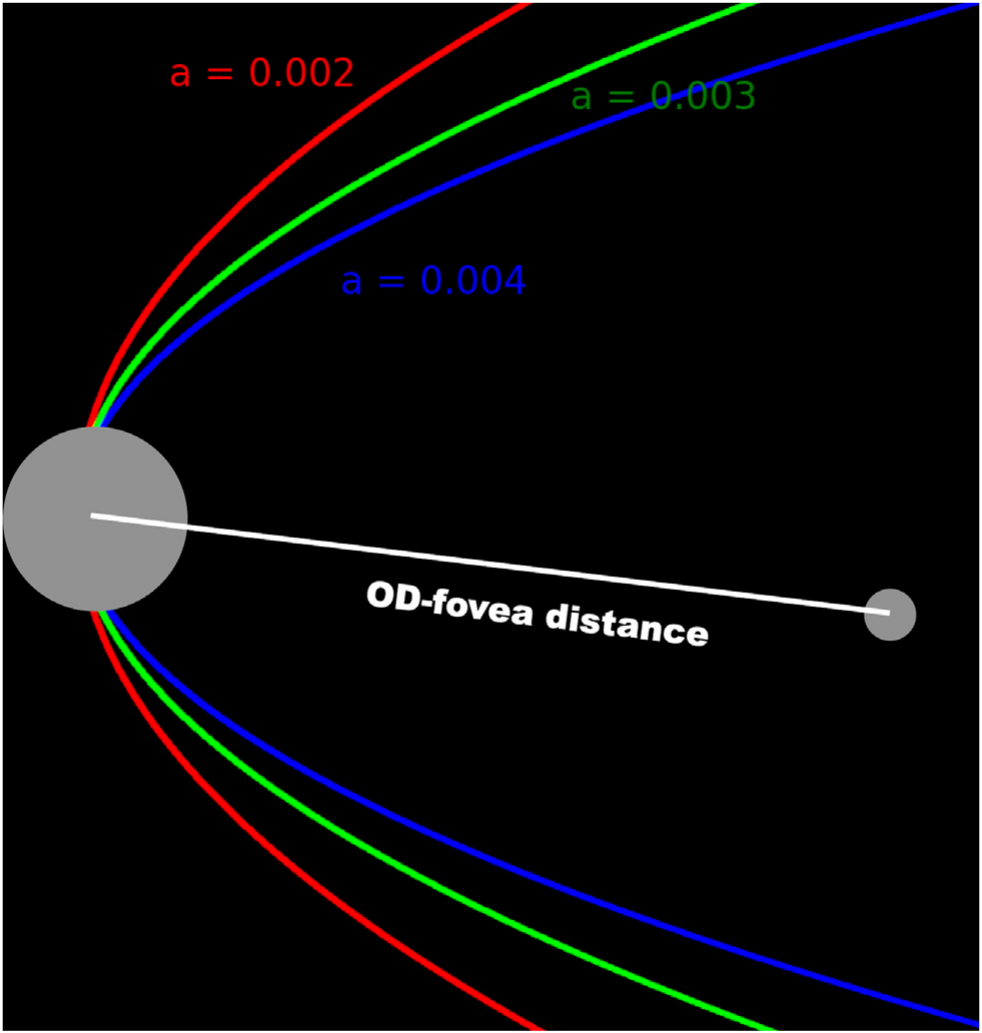
Optic disc–fovea distance is the Euclidean (shortest) distance from the center of the OD to the center of the foveal region. Temporal arterial/venous concavity describes the parabolic course (or the degree of convergence) of the temporal artery/vein. A larger value (denoted by “a”) indicates that the temporal arterial/venous arcade curves more inwardly toward the fovea (blue curve), whereas a smaller value indicates a smaller degree of convergence (green or red curve). OD = optic disc.

Optic disc–fovea distance, temporal arterial concavity, and temporal venous concavity (Fig 2) were derived using fully automated pipelines detailed previously.^13^ Optic disc–fovea distance was the Euclidian (shortest) distance from the center of the OD to the center of the foveal region, corrected for the influence of ocular magnification. Temporal arterial or venous concavity was a dimensionless metric describing the parabolic course (i.e., degree of convergence) of the temporal retinal artery or vein, respectively. A larger concavity value indicated that the temporal arterial/venous arcade curved more inwardly toward the fovea.

### Statistical Analysis

The association between spherical equivalent refraction (SER) and NRR pallor was tested both globally and by sector—with age, sex, ethnic group (White or non-White), IOP, and mean arterial pressure included as potential covariates. To determine whether the effect of SER on pallor varied between ethnic groups, an interaction term between SER and ethnic group was also added. After this, the influence of OD–fovea distance and temporal arterial/venous concavity on sectoral NRR pallor was examined by incorporating them (simultaneously) as additional independent variables into the models above, but without the interaction term. Linear mixed-effects models were fitted in all instances, with individuals treated as random effects to account for intereye correlation, using the *lmerTest* package in R version 4.2.2 (R Core Team 2022).^21^

The “White” ethnic group was defined as those who were self-reportedly “British” (n = 20 006), “Irish” (n = 786), “White” (n = 20), or of “any other White background” (n = 1236), while the “non-White” group included “Indian” (n = 456), “Caribbean” (n = 356), “other ethnic group” (n = 341), “African” (n = 250), “any other Asian background” (n = 139), “Chinese” (n = 100), “Pakistani” (n = 92), “White and Asian” (n = 77), “any other mixed background” (n = 76), “White and Black Caribbean” (n = 58), “White and Black African” (n = 41), “Bangladeshi” (n = 14), “any other Black background” (n = 6), “Mixed” (n = 2), “Black or Black British” (n = 1), and “Asian or Asian British” (n = 0). The decision to classify all “non-White” individuals as a single entity was motivated by the disproportionately low numbers within each “non-White” ethnicity, as the ethnic composition of the UK Biobank is predominantly White, reflecting the demographics of the United Kingdom. Mean arterial pressure was calculated as 0.33 × systolic blood pressure + 0.67 × diastolic blood pressure.^22^ The source code for all regression analyses described herein is available at github.com/fyii200/MyopicRimPallor.

## Results

**Figure 3:**
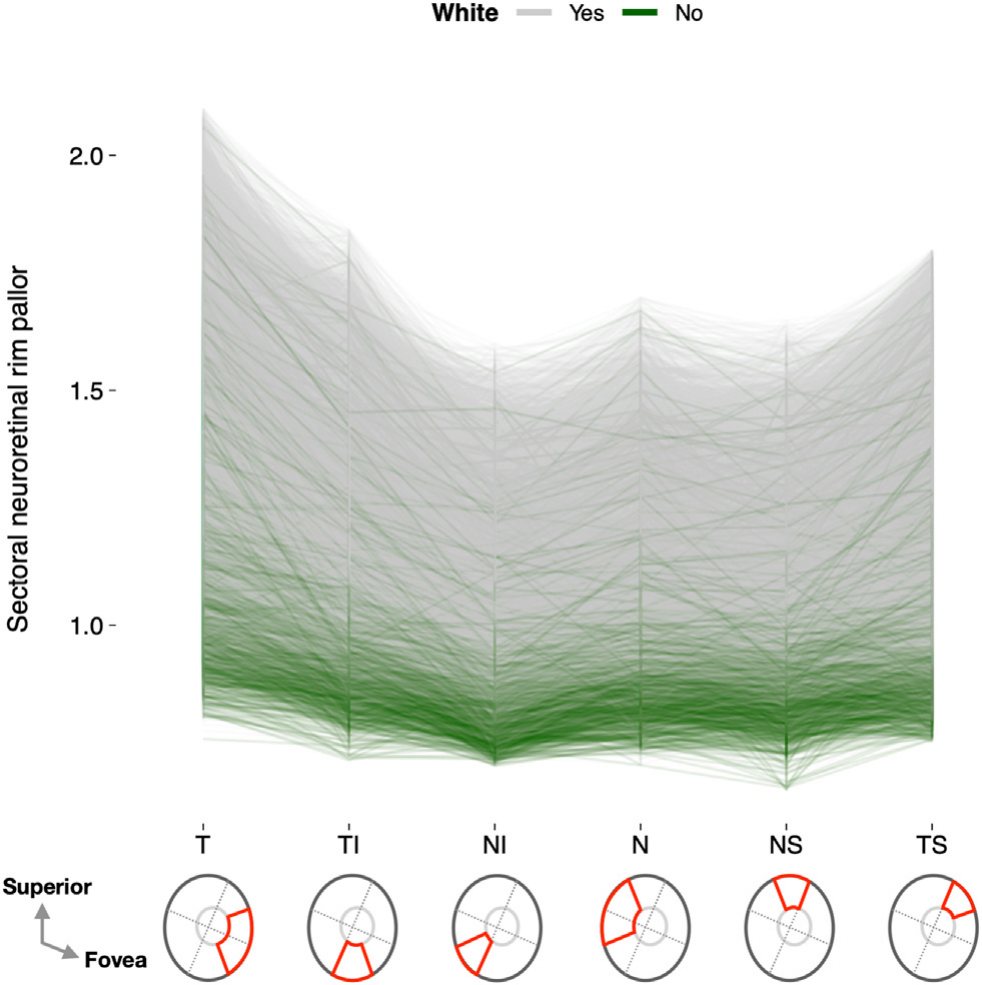
Neuroretinal rim pallor profile across the temporal (T), temporal inferior (TI), nasal inferior (NI), nasal (N), nasal superior (NS), and temporal superior (TS) sectors by ethnic group.

Table 1 summarizes the characteristics of the included participants and eyes, while Figure 3 displays the profile of NRR pallor in individual eyes across all 6 sectors. As shown in the figure, moving clockwise from the temporal sector to the temporal superior sector, NRR pallor generally varied in an asymmetrical “U” shape: it peaked in the temporal sector, decreased toward the more nasal sectors, and then increased slightly again in the temporal superior sector. While this U-shaped profile was evident in both ethnic groups, White participants appeared to have higher NRR pallor on average across all sectors.

**Table 1:** Characteristics of Included Participants and Eyes

Characteristic	All	White	Non-White
Eyes (participants)	33 950 (24 057)	31 319 (22 243)	2917 (2009)
Female	13 927 (57.9%)	12 775 (57.9%)	1163 (57.3%)
Age, yrs	53.5 (8.1)	53.9 (8.1)	48.9 (7.0)
SER, D	−0.47 (2.25)	−0.46 (2.27)	−0.68 (2.15)
IOP, mmHg	15.6 (3.7)	15.7 (3.8)	14.8 (3.8)
MAP, mmHg	97.6 (11.2)	97.6 (11.2)	97.1 (11.8)
NRR Pallor			
Global	1.24 (0.20)	1.26 (0.20)	1.05 (0.17)
Temporal	1.40 (0.28)	1.42 (0.27)	1.17 (0.23)
Temporal inferior	1.22 (0.21)	1.24 (0.20)	1.02 (0.17)
Nasal inferior	1.08 (0.16)	1.10 (0.16)	0.91 (0.14)
Nasal	1.14 (0.17)	1.16 (0.16)	0.97 (0.15)
Nasal superior	1.12 (0.16)	1.14 (0.15)	0.95 (0.14)
Temporal superior	1.22 (0.22)	1.24 (0.22)	1.03 (0.19)

D = diopter; IOP = intraocular pressure; MAP = mean arterial pressure; NRR = neuroretinal rim; SER = spherical equivalent refraction.

Mean (standard deviation) shown for continuous variables.

Table 2 shows the effect of SER on NRR pallor while controlling for age, sex, ethnic group, IOP, and mean arterial pressure. A significant negative association between SER and pallor was evident both globally and in all 6 NRR sectors. There was insufficient evidence of an interaction effect between SER and ethnic group on either global or sectoral pallor, indicating no ethnic difference in the direction or magnitude of the association between SER and NRR pallor. However, White participants had much paler NRR on average (about 0.20–0.28 difference globally and by sector), confirming the observation from Figure 3. Likewise, there was statistical evidence of higher NRR pallor in males compared with females, but this sex difference was negligible (approximately 0.01 only). Younger age and higher mean arterial pressure were associated with higher NRR pallor, both globally and by sector.

**Table 2:** Association of SER with Neororetinal Rim Pallor (Globally and by Sector) and Optic Disc Pallor (Globally), Controlling for Age, Sex, Glaucoma Status, IOP, and MAP

Variable	Neuroretinal Rim Pallor						
	Global	Temporal	TI	NI	Nasal	NS	TS
	ß [95% CI]	ß [95% CI]	ß [95% CI]	ß [95% CI]	ß [95% CI]	ß [95% CI]	ß [95% CI]
Intercept	1.454 [1.431, 1.478]∗∗∗	1.732 [1.700, 1.764]∗∗∗	1.419 [1.395, 1.443]∗∗∗	1.164 [1.146, 1.183]∗∗∗	1.265 [1.245, 1.285]∗∗∗	1.235 [1.216, 1.254]∗∗∗	1.453 [1.427, 1.479]∗∗∗
SER	−0.006 [–0.007, −0.005]∗∗∗	−0.009 [–0.011, −0.008]∗∗∗	−0.008 [–0.009, −0.007]∗∗∗	−0.004 [–0.005, −0.004]∗∗∗	−0.003 [–0.003, −0.002]∗∗∗	−0.005 [–0.006, −0.004]∗∗∗	−0.004 [–0.005, −0.003]∗∗∗
NW	−0.235 [–0.244, −0.227]∗∗∗	−0.278 [–0.289, −0.266]∗∗∗	−0.236 [–0.245, −0.227]∗∗∗	−0.195 [–0.202, −0.188]∗∗∗	−0.204 [–0.211, −0.197]∗∗∗	−0.205 [–0.212, −0.198]∗∗∗	−0.232 [–0.242, −0.222]∗∗∗
SER × NW	−0.0003 [–0.004, 0.003]	−0.0002 [–0.005, 0.005]	−0.001 [–0.005, 0.003]	0.001 [–0.002, 0.003]	0.001 [–0.002, 0.004]	−0.001 [–0.004, 0.002]	−0.001 [–0.005, 0.004]
Age	−0.004 [–0.005, −0.004]∗∗∗	−0.007 [–0.007, −0.006]∗∗∗	−0.004 [–0.004, −0.004]∗∗∗	−0.002 [–0.003, −0.002]∗∗∗	−0.003 [–0.003, −0.003]∗∗∗	−0.003 [–0.003, −0.002]∗∗∗	−0.005 [–0.005, −0.005]∗∗∗
Male	0.010 [0.006, 0.015]∗∗∗	0.010 [0.004, 0.016]∗∗	0.010 [0.005, 0.014]∗∗∗	0.012 [0.008, 0.016]∗∗∗	0.010 [0.006, 0.014]∗∗∗	0.011 [0.008, 0.015]∗∗∗	0.011 [0.006, 0.016]∗∗∗
IOP	−0.0004 [–0.001, 0.0002]	−0.0003 [–0.001, 0.001]	−0.0004 [–0.001, 0.0002]	−0.0002 [–0.001, 0.0003]	−0.001 [–0.001, −0.0001]∗	−0.0003 [–0.001, 0.0001]	−0.0002 [–0.001, 0.0005]
MAP	0.0004 [0.0001, 0.001]∗∗∗	0.0003 [0.00003, 0.001]∗	0.0003 [0.0001, 0.0005]∗	0.0005 [0.0003, 0.001]∗∗∗	0.0005 [0.0003, 0.001]∗∗∗	0.0004 [0.0002, 0.001]∗∗∗	0.0005 [0.0002, 0.001]∗∗∗

IOP = intraocular pressure; MAP = mean arterial blood pressure; NI = nasal inferior; NS = nasal superior; NW = non-White; SER = spherical equivalent refraction; TI = temporal inferior; TS = temporal superior; ß = unstandardized beta coefficient for nonintercept terms (in bold); 95% CI = 95% confidence interval.

∗∗∗*P* ≤ 0.0001; ∗∗∗*P* < 0.001; ∗*P* < 0.05. All values are rounded to 3 decimal places or to the nearest decimal.

**Figure 4:**
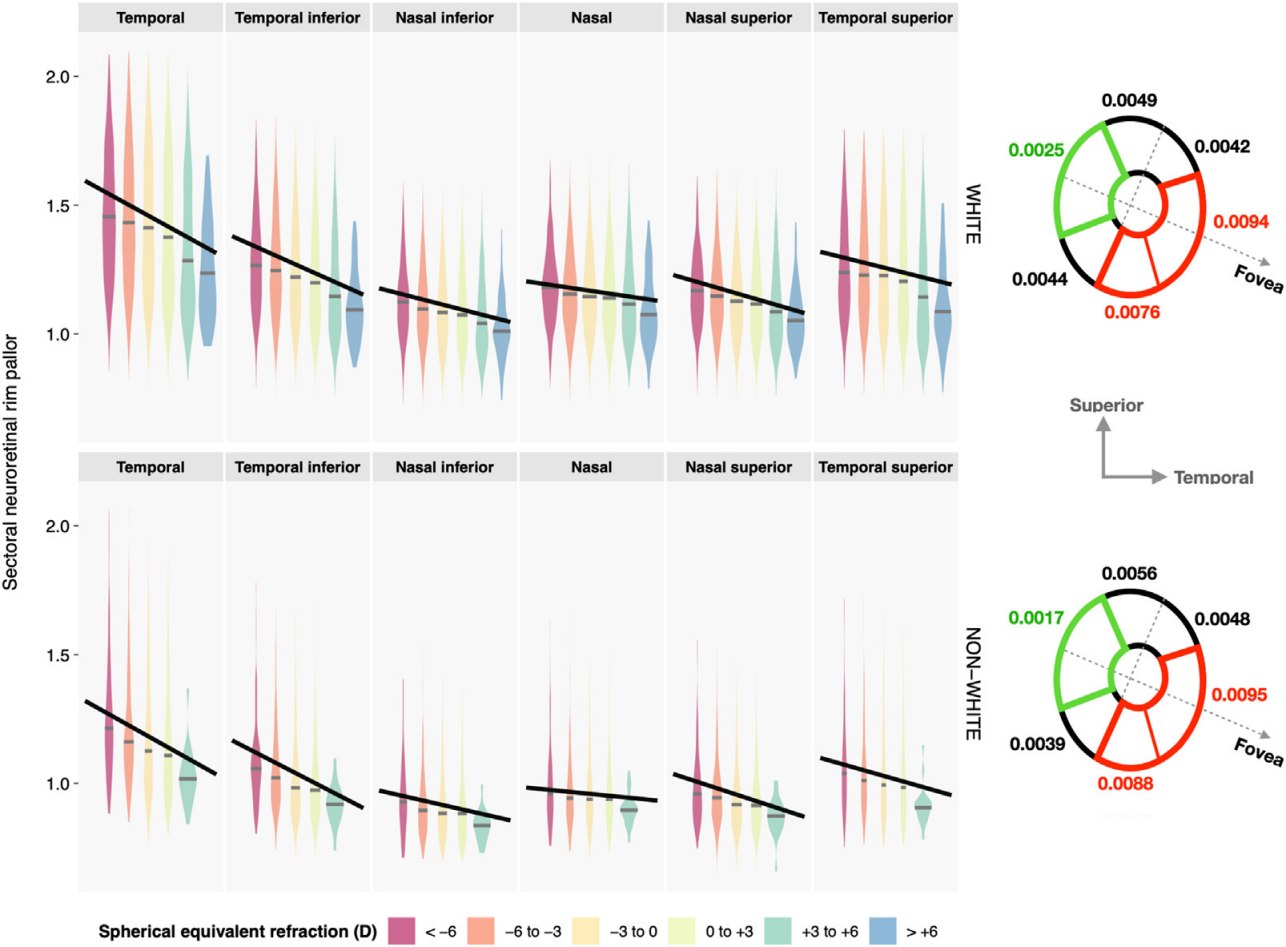
Changes in sectoral neuroretinal rim pallor from high myopia through to high hyperopia in White (top) and non-White (bottom) participants. Regression lines are based on the model shown in Table 2, which controlled for age, sex (lines shown are specific to females), intraocular pressure, and mean arterial pressure. The different neuroretinal rim sectors (right) are color-coded according to the amount of increase in pallor per diopter (D) increase in refractive error in the myopic direction (steepness of the regression lines): red indicates the steepest change, followed by black, and finally green.

While pallor increased with decreasing SER in all NRR sectors, there were clear differences in the rate of change among the different sectors, irrespective of ethnic group (Fig 4 and Table 2). Specifically, the temporal and temporal inferior sectors (red NRR zone in Fig 4) exhibited the steepest changes with refractive error, where pallor increased by approximately 0.009 and 0.008 for every diopter increase in SER in the myopic direction. The rate of change reduced by a factor of 2 toward the more nasal sectors (black NRR zone) and by another 2 to around 0.0025 in the nasal-most sector (green NRR zone). Of note, there was no overlap among the 95% confidence intervals for the SER regression coefficients in the red (temporal and temporal inferior), green (nasal), and black (nasal inferior, nasal superior, and temporal superior) zones (Table 2), suggesting significant zonal differences in the rate of change.

The observation that NRR pallor increased at much higher rates temporally than nasally aligns with the varying effects of OD–fovea distance and temporal vessel concavity on pallor across different NRR sectors. As indicated by the regression analysis in Table 3, each unit increase in OD–fovea distance resulted in a 0.004 increase in pallor in the temporal sector, independently of SER. This increase was 4 times larger than the 0.001 increase observed in the nasal NRR, with no overlap between their 95% confidence intervals (0.003–0.004 vs. 0.001–0.001). Likewise, NRR pallor increased by 5.1 and 9.0 units for every unit increase in temporal arterial and venous concavity in the temporal NRR, respectively. These increases were much larger than those observed in any other sector—particularly the nasal sector—where the corresponding estimates were more than twice as small at 1.41 for arterial concavity and 3.80 for venous concavity. Comparing the beta coefficients for SER in Table 2 with those in Table 3, the inclusion of OD–fovea distance and arterial/venous concavity weakened the association between SER and pallor by around ≥50% across all NRR sectors, suggesting that the effect of SER on NRR pallor was attributable in large part to changes in these retinal features.

**Table 3:** Association of OD–Fovea Distance and Temporal Arterial/Venous Concavity with Sectoral Neuroretinal Rim Pallor, Controlling for SER, Ethnic Group, Age, Sex, Intraocular Pressure, and Mean Blood Pressure

Variable	Neuroretinal Rim Pallor					
	Temporal	TI	NI	Nasal	NS	TS
	ß [95% CI]	ß [95% CI]	ß [95% CI]	ß [95% CI]	ß [95% CI]	ß [95% CI]
OD–fovea distance	0.004 [0.003, 0.004]∗∗∗	0.002 [0.002, 0.002]∗∗∗	0.001 [0.001, 0.002]∗∗∗	0.001 [0.001, 0.001]∗∗∗	0.001 [0.001, 0.001]∗∗∗	0.003 [0.002, 0.003]∗∗∗
Concavity						
Temporal artery	5.07 [1.36, 8.77]∗∗	2.93 [0.17, 5.70]∗	3.00 [0.80, 5.20]∗∗	1.41 [–0.85, 3.68]	2.16 [–0.01, 4.33]	4.20 [1.17, 7.22]∗∗
Temporal vein	9.01 [6.09, 11.93]∗∗∗	6.49 [4.32, 8.67]∗∗∗	5.43 [3.70, 7.17]∗∗∗	3.80 [2.02, 5.58]∗∗∗	4.50 [2.80, 6.21]∗∗∗	7.34 [4.96, 9.72]∗∗∗
SER	−0.003 [–0.005, −0.002]∗∗∗	−0.004 [–0.005 to −0.003]∗∗∗	−0.002 [–0.002 to −0.001]∗∗∗	0.0002 [–0.001 to 0.001]	−0.002 [–0.003 to −0.001]∗∗∗	0.0004 [–0.001 to 0.002]

NI = nasal inferior; NS = nasal superior; OD = optic disc; SER = spherical equivalent refraction; TI = temporal inferior; TS = temporal superior; ß = unstandardized beta coefficient; 95% CI = 95% confidence interval.

Estimates for OD–fovea distance, temporal arterial/venous concavity, and SER are displayed.

∗∗∗*P* ≤ 0.0001; ∗∗∗*P* < 0.001; ∗*P* < 0.05.

## Discussion

Progressing clockwise from the temporal sector through to the temporal superior sector (Fig 3), NRR pallor varies in an asymmetrical U-shaped pattern (paler temporally than nasally). This corresponds to the n-shaped thickness profile of the NRR, which is thinner temporally than nasally.^23^ Increasing myopia or decreasing hyperopia leads to greater pallor in all 6 NRR sectors. However, the rate of change differs significantly among these sectors (Fig 4), with the temporal and temporal inferior NRR exhibiting the largest increase in pallor per diopter change in refractive error. This increase diminishes by a factor of 2 toward the more nasal/central sectors, and by another 2 in the nasal-most sector. In line with this, increasing OD–fovea distance and temporal arterial/venous concavity result in more than twice (and up to 4 times) as much NRR pallor temporally compared with nasally. These retinal changes account for approximately ≥50% of the effect of refractive error on NRR pallor. While White participants tend to have paler NRR, there is no ethnic difference in the association between SER and pallor in any of the 6 NRR sectors. However, a limitation of the present work is the inability to subcategorize non-White participants into their respective ethnicities due to their low numbers in the UK Biobank.

No previous studies have empirically examined changes in NRR pallor across refractive error, although the disc/NRR is generally believed to appear paler in myopia.^10^^,^^24^^,^^25^ The increase in NRR pallor with decreasing SER observed in this work may have a mechanical or vascular etiology, or a combination of both.^26^ First, the impact of myopic ocular expansion on ONH morphology may directly influence the degree of perceived pallor in fundus photography. OCT-derived minimum rim thickness—defined^27^ as the smallest distance from the true anatomical border of the NRR (which is Bruch's membrane opening [BMO]) to the internal limiting membrane—has been shown to decrease in high myopia (mean SER: −7.61 diopters),^3^ coinciding with a (potential) decrease in the thickness of the peripapillary retinal nerve fiber layer.^28^ This suggests some degree of prelaminar axonal loss in eyes with high myopia, which likely affects light reflectance from the NRR tissue (perhaps by increasing light transmission to the underlying lamina cribrosa).^26^^,^^29^ Whether a similar change in minimum rim thickness occurs in low-to-moderate myopia, or from hyperopia to emmetropia, remains to be corroborated because pertinent research is limited,^3^ and an earlier study did not find such evidence in eyes with lower levels of myopia (mean SER: −1.89 diopters).^28^

**Figure 5:**
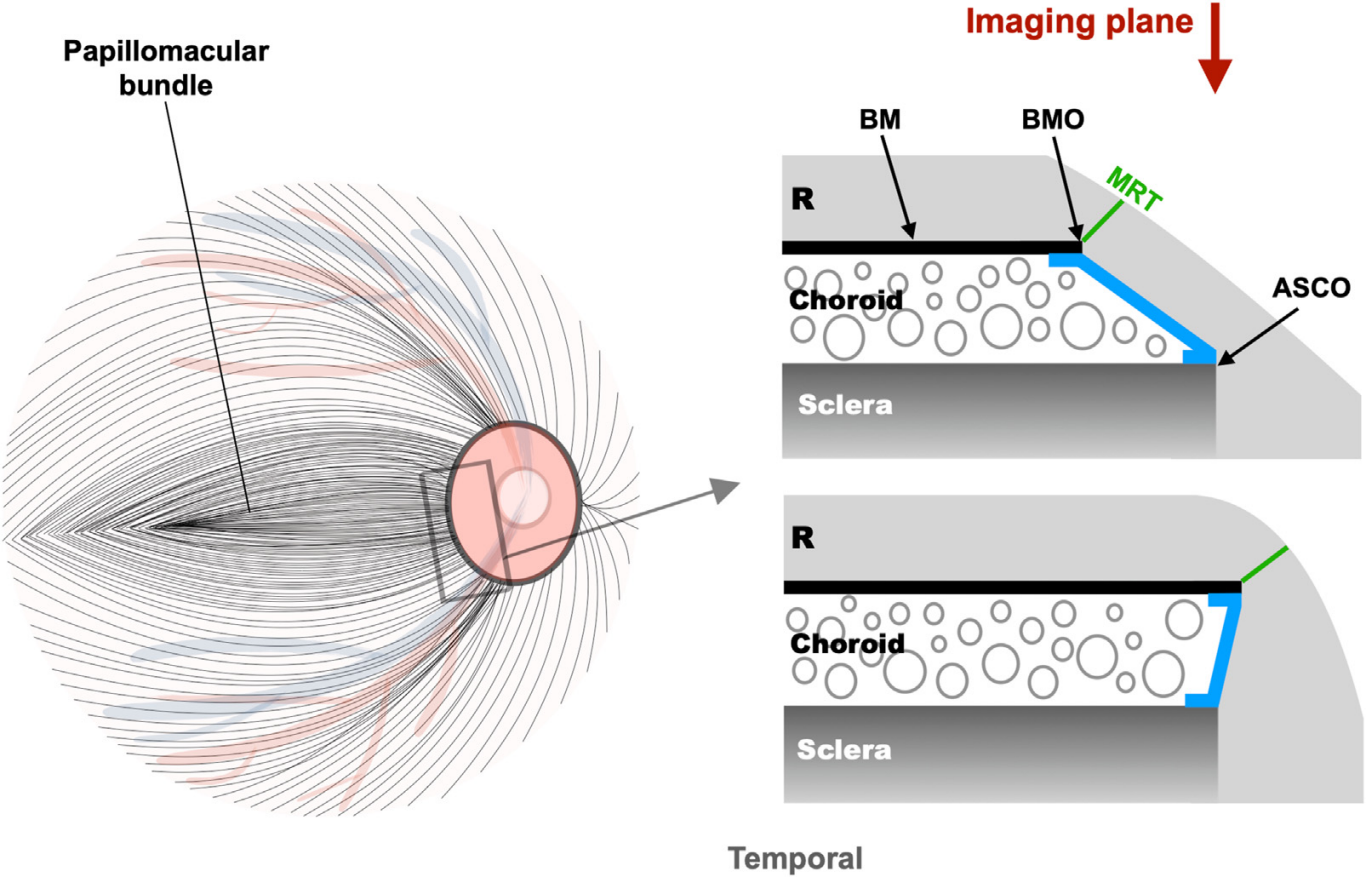
In an axially elongated eye (top right), a relative temporal shift of the Bruch's membrane (BM) causes the neuroretinal rim border tissue (blue region) to extend from the anterior scleral canal opening (ASCO) to the BM opening (BMO) in a centrifugal direction, away from the optic nerve head (ONH), particularly in the temporal/inferior region. This externally oblique rim border configuration causes the retinal nerve fibers (R) to spread over a wider ONH region perpendicular to the imaging plane (i.e., horizontally), thereby reducing rim thickness along the imaging plane more than that seen in an internally oblique configuration (bottom right)—irrespective of whether there is a difference in minimum rim thickness (MRT). In addition to this morphological change in rim border tissue, the papillomacular nerve fiber bundle (left), which connects the macula to the temporal rim, may be more susceptible to mechanical damage from myopic stretching along the disc–fovea axis. This increased vulnerability arises because the papillomacular bundle follows a relatively straight course, potentially limiting its ability to adapt to the increasing disc–fovea distance and temporal arterial/venous concavity seen in eyes with more negative/myopic refractive error. In contrast, the more peripherally located nerve fibers, which are connected to nontemporal rim sectors, follow a more arcuate course and can therefore better adapt by straightening. These changes—coupled with reduced ONH blood perfusion, which is more pronounced temporally than nasally—may collectively explain why pallor increases much more rapidly in the temporal and temporal inferior rim sectors with decreasing spherical equivalent refraction.

Another related mechanical explanation points to an increased tendency for the NRR border tissue to adopt an externally oblique configuration in eyes undergoing axial elongation (Fig 5).^30^ In such eyes, a temporal displacement of BMO relative to the anterior scleral canal opening causes the NRR border tissue to extend from the anterior scleral canal opening to BMO in a centrifugal direction away from the ONH, especially in the temporal/inferior region.^5^^,^^8^^,^^31^ Since ganglion cell axons must traverse the BMO before passing through the anterior scleral canal opening, an increasing tendency for the temporal/inferior NRR border tissue to be oriented externally obliquely means that in eyes with higher myopia, the axons must spread over a wider area in a direction opposite to the imaging plane. This causes NRR thickness along the imaging plane to decrease more in the temporal/inferior NRR compared with the more nasal sectors—even in the absence of a change in minimum rim thickness—potentially explaining the steeper increase in temporal and temporal inferior NRR pallor with decreasing SER (Fig 5). In addition to these mechanical explanations, increased NRR pallor in myopia may reflect decreased ONH blood perfusion.^26^ In support of this, the density of radial peripapillary capillaries—or the microvasculature supplying the ganglion cell axons at the ONH—has been shown to decrease with decreasing SER or increasing axial length using OCT angiography.^32^, ^33^, ^34^, ^35^ Of note, this reduction is more pronounced temporally than nasally,^32^ fitting with the observation of a faster change in NRR pallor temporally than nasally as SER decreases.

A myopia-related temporal/inferior shift of the BMO (as mentioned above) indicates that axial elongation stretches the ONH border predominantly in the temporal direction along the OD–fovea axis. Such a BMO shift, seen fundoscopically as the formation or enlargement of parapapillary gamma zone inferotemporally,^4^ accounts for an increase in the OD–fovea distance and a decrease in the so-called angle kappa (related to increased concavity) of the temporal retinal vessels in high myopia.^14^^,^^16^^,^^36^ These fundal changes are consequently believed to reflect a greater degree of axonal stretching at the posterior pole.^9^^,^^12^^,^^16^ For example, an increased temporal convergence of retinal nerve fiber bundles^37^ in eyes with higher myopia is accompanied by a similar change in the temporal retinal vessels (i.e., increased concavity).^13^ On this note, the papillomacular retinal nerve fibers run relatively straight from the temporal NRR to the macula (Fig 5), so they have been suggested to be more susceptible to mechanical damage from myopic stretching.^12^ The more peripherally located nerve fibers, in contrast, follow a more arcuate course and can therefore better adapt to such stretching by straightening.^12^ Our observation that the temporal NRR, which contains the papillomacular bundle, exhibits the steepest increase in pallor with increasing OD–fovea distance and vessel concavity supports the notion that the papillomacular bundle is more susceptible to posterior pole stretching than the arcuate bundle. This may also be another reason why decreasing SER, which stretches the papillomacular bundle through its association with increasing OD–fovea distance and vessel concavity,^13^ leads to the steepest increase in NRR pallor temporally.

As noted earlier, White ethnicity was associated with increased NRR pallor. It is unclear if this ethnic effect reflects intrinsic differences in ONH parameters, given that White ethnicity has been reported to be associated with a reduction in peripapillary nerve fiber layer thickness,^38^, ^39^, ^40^, ^41^, ^42^, ^43^ but an increase in minimum rim thickness,^39^^,^^44^ compared with Asians or Africans. An alternative explanation points to the influence of background retinal pigmentation (significantly more pronounced in non-White individuals)^45^ on the luminosity of the NRR, as the normally pinkish-orange NRR derives its color not only from the capillaries within the ONH but also from the incident light scattered from the surrounding choroid and retina.^46^ Regardless of the underlying reason, the significant effect of ethnicity on NRR pallor in normal eyes—independent of refractive error and other potential confounders—suggests that clinicians should be aware of the normative difference in NRR pallor between White and non-White patients when using it as a diagnostic criterion for conditions such as myopia-related NGON.^9^ Similar to ethnicity, it is unclear if the age-related reduction in NRR pallor observed in this study is attributable to changes in ONH parameters, as minimum rim thickness and peripapillary nerve fiber layer thickness generally decrease with older age.^39^, ^40^, ^41^, ^42^, ^43^, ^44^ Age-related cataract is also unlikely to be the cause, given that only eyes with good visual acuity were included. Instead, we suggest that senile miosis may play a role by attenuating the exposure of fundus photographs,^47^ although further research is needed to investigate the effect of pupil diameter on NRR pallor. No research has looked at the influence of blood pressure on NRR pallor, but the positive association observed in Table 2 adds to recent findings of a positive relationship between systolic blood pressure and NGON risk in highly myopic eyes.^9^

In summary, NRR pallor increases with decreasing SER in normal eyes. The rate of increase is highest within the temporal and temporal inferior NRR, coinciding with the typical initial location of glaucomatous damage,^48^ which compounds the difficulty of detecting glaucoma in myopic eyes.^25^ Moving nasally from these temporal sectors along the disc–fovea axis, the rate of increase diminishes by half toward the more central NRR and by another half in the nasal-most NRR. Consistent with these changes, increasing disc–fovea distance and temporal arterial/venous concavity result in twofold to fourfold steeper increase in NRR pallor temporally compared with nasally. These retinal changes account for approximately half or more of the effect of refractive error on NRR pallor. Collectively, our findings support the notion that the papillomacular nerve fiber bundle, which follows a relatively straight course from the temporal NRR to the macula, is most susceptible to mechanical damage from myopic stretching.

## Data Availability

This research was conducted using data from the UK Biobank under project ID 90655. Data directly supporting the results of this work are only available to the immediate research team members due to UK Biobank's access control policy. Bona fide researchers can, however, apply for access at ukbiobank.ac.uk/enable-your-research/apply-for-access. Source code used to perform the regression analyses described in this work is freely available at github.com/fyii200/MyopicRimPallor. The automated software used to derive neuroretinal rim pallor is available upon reasonable request.
