# Supplementary material for: Sectoral Changes in Neuroretinal Rim Pallor Across Refractive Error
Source: Ophthalmol Sci. 2025 Jan 7;5(3):100705. doi: 10.1016/j.xops.2025.100705 (PMC11938055; doi:10.1016/j.xops.2025.100705)
Supplement: Supplementary Material S1 and S2 [file mmc1.pdf]

S1: Automated quality control

The PallorMetrics software[1] outputs a range of continuous metrics, including neuroretinal rim pallor (both globally and across different sectors), disc crowdedness, disc wobbliness, disc-fovea distance, disc eccentricity, vessel mass and control region intensity (Table 1). In addition to these metrics, the software generates visualisations of the segmentations and transformations that occur during image processing, allowing the user to assess whether an image should be rejected due to suboptimal processing caused by poor image quality. Here, we describe a method to automate this process by applying rejection thresholds to the software's various outputs.

Table 1. Description of various metrics output by the PallorMetrics software.

Metric	Description
Pallor	
Global	Pallor across the whole measurement region in the neuroretinal rim.
Temporal	Pallor in the temporal sector of the neuroretinal rim.
Temporal-inferior	Pallor in the temporal-inferior sector of the neuroretinal rim.
Nasal-inferior	Pallor in the nasal-inferior sector of the neuroretinal rim.
Nasal	Pallor in the nasal sector of the neuroretinal rim.
Nasal-superior	Pallor in the nasal-superior sector of the neuroretinal rim.
Temporal-superior	Pallor in the temporal-superior sector of the neuroretinal rim.
Nasal-temporal ratio	Nasal neuroretinal rim pallor divided by temporal neuroretinal rim pallor.
Disc crowdedness	The ratio of vessel pixels inside the disc to the area of the disc. A value close to 0 indicates an unusually low density of disc vessels, likely due to poor segmentation of the vessel and/or disc.
Disc wobbliness	The intersection over union between the segmented disc and the best-fitting ellipse. A very small value suggests an unusually distorted shape, likely due to poor disc segmentation.
Disc-fovea distance	Distance in pixels from the temporal edge of the disc to the fovea. An unusually small value is a sign of incorrect localisation of the disc and/or fovea.
Disc eccentricity	Distance between the two foci of the best-fitting ellipse divided by its major axis length, where 0 is a perfect circle and 1 is a line. A value close to 1 is therefore very likely due to poor disc segmentation.
Vessel mass	Total number of vessel pixels inside the cropped square around the disc. An unusually small value therefore suggests poor vessel segmentation.
Control region intensity	Median pixel intensity in the control region. An unusually small or large value suggests severe underexposure or overexposure of the image.

Determination of optimal rejection thresholds for different continuous metrics

The first ~100 colour fundus photographs from each of the 12 datasets detailed in Table 2, including the UK Biobank, were sampled, resulting in a total of 1,485 images. These images were used to determine the optimal rejection thresholds for each of the continuous metrics listed in Table 1, following the method described below.

Table 2. Details of the 12 datasets—representing a diverse range of eye conditions (normal and diseased), image resolutions, fundus cameras and populations—used to derive the rejection thresholds for the various metrics output by the software.

Dataset	Description	Number	Format	Resolution (pixels)
UK Biobank[2]	Population-based cohort used in the present study (UK).	104	PNG	2048×1536
ORIGA[3]	Normal and glaucomatous eyes (Singapore).	99	JPG	2495×2048
MESSIDOR[4]	Normal eyes and eyes with diabetic retinopathy (France).	100	PNG	2240×1488
PREVENT Dementia[5]	Mid-life adults at increased risk of dementia (UK).	100	PNG	3072×2048
Kaggle cataract dataset[6]	Unclear provenance, but data provider is affiliated with a university in South Korea at the time of writing.			
Normal		100	PNG	mixed
Glaucoma		100	PNG	mixed
Cataract		100	PNG	mixed
Retinal disease		100	PNG	mixed
IDRiD[7]	Normal eyes and eyes with diabetic retinopathy (India).	81	PNG	4288×2848
LBC 1936[8]	Elderly (all aged around 72 years) healthy cohort (UK).	100	PNG	2689×2186
PAPILA[9]	Normal and glaucomatous eyes (Spain).	100	JPG	2576×1934
G1020[10]	Normal and glaucomatous eyes (Germany).	100	JPG	mixed
FIVES[11]	Normal eyes and eyes with conditions including glaucoma, and age-related macular degeneration (China).	100	PNG	2048×2048
Kaggle ODIR-5K[12]	Normal eyes and eyes with conditions such as glaucoma, cataract, age-related macular degeneration and pathologic myopia (China).	101	JPG	2592×1728
RFMiD[13]	Normal eyes and eyes with a range of conditions or pathological findings such as age-related macular degeneration and central retinal artery/vein occlusion (India)	100	PNG	2144×1424

The 1,485 sampled images were first processed using the software. Of these, 4 images could not be processed due to significant pathology or media opacity that obscured all or most of the optic disc, fovea and retinal vessels (Figure 1). An experienced image grader (SG) then manually labelled each of the remaining 1,481 images as either “keep” or “reject” by carefully inspecting the quality of the segmentations generated by the software based on the following criteria: (1) correct segmentation and localisation of the optic disc; (2) correct localisation of the fovea; and (3) correct segmentation of the retinal vessels. Based on these criteria, 326 images were marked as “reject” (22.0%). Examples of images labelled as “keep” and “reject” are shown in Figures 2 and 3.

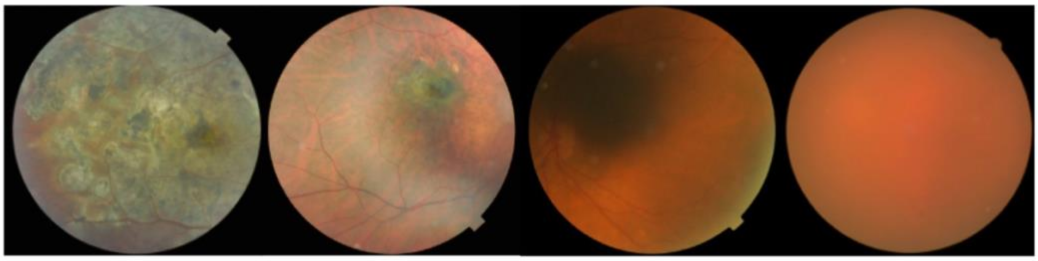

Figure 1. Four images could not be processed by the software due to significant pathology or media opacity obscuring all or most of the optic disc, fovea and retinal vessels.

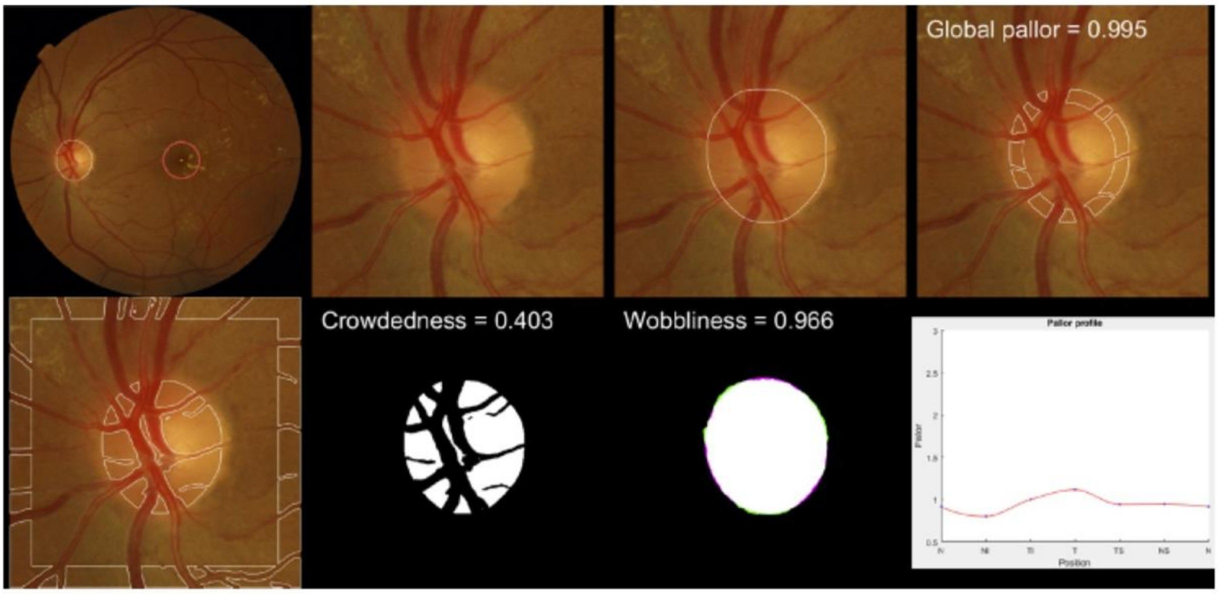

Figure 2. Example of a processed image labelled as "keep". The optic disc, fovea and retinal vessels are correctly localised and segmented, allowing neuroretinal rim pallor to be accurately derived from the correct measurement region within the optic nerve head while disregarding the vessel pixels.

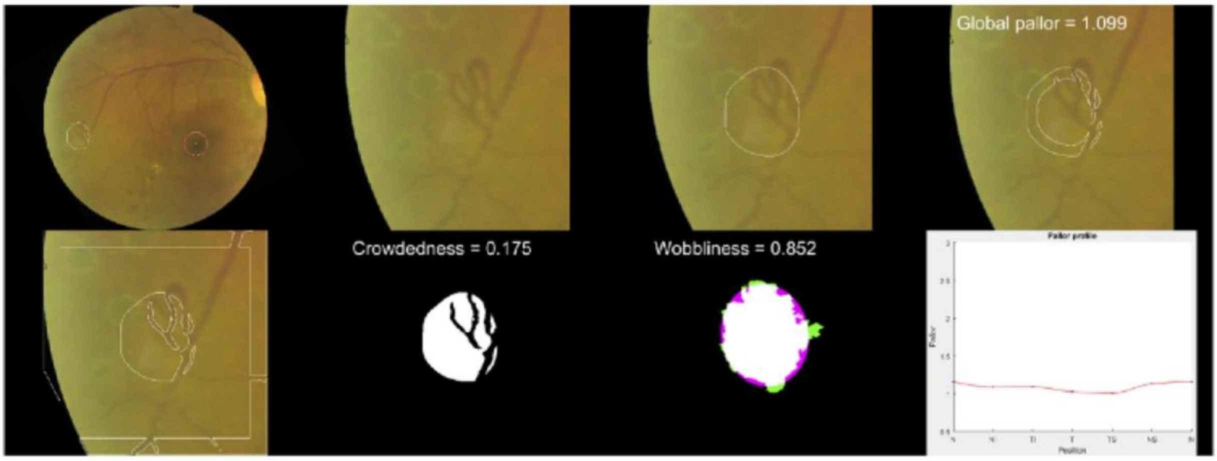

Figure 3. Example of a processed image labelled as "reject". A large part of the optic disc is hidden due to poor alignment during image acquisition, causing inaccurate disc localisation and segmentation.

Rejection thresholds for various metrics (Table 3) were empirically determined by visualising their respective distributions, stratified by the ground-truth image quality labels obtained above. Specifically, for each metric, we identified the optimal cut-off value(s) that would capture the greatest number of “reject” datapoints while minimising overlap with the “keep” datapoints in the distribution (i.e. manually determining the best boundary to separate the “keep” classes from the “reject” classes).

Table 3. Thresholds for deciding whether a processed image should be kept or rejected. For a given metric, a value falling outside the specified thresholds can be considered so extreme that it is likely due to suboptimal processing caused by poor image quality, rather than normal variations or eye condition(s).

Metric	Thresholds	Number of images outside thresholds
Pallor		
Global	0.75 – 1.95	6 (0.4%)
Temporal	0.75 – 2.1	9 (0.6%)
Temporal-inferior	0.7 – 1.85	7 (0.5%)
Nasal-inferior	0.7 – 1.6	12 (0.8%)
Nasal	0.7 – 1.7	9 (0.6%)
Nasal-superior	0.65 – 1.65	6 (0.4%)
Temporal-superior	0.75 – 1.8	7 (0.5%)
Nasal-temporal ratio	0.6 – 1.15	22 (1.5%)
Disc crowdedness	< 0.25	73 (4.9%)
Disc wobbliness	< 0.92	178 (12.0%)
Disc-fovea distance	< 330 pixels	19 (1.0%)
Disc eccentricity	> 0.75	4 (0.3%)
Vessel mass	< 52,000 pixels	58 (4.0%)
Control region intensity	0.2 – 1.0	4 (0.3%)

Based on the rejection thresholds derived above, an image would be automatically predicted as "reject" if any of its metrics fell outside the specified thresholds. The confusion matrix and performance metrics based on this method are presented in Figure 4 and Table 4, showing very high sensitivity and specificity (around 90%).

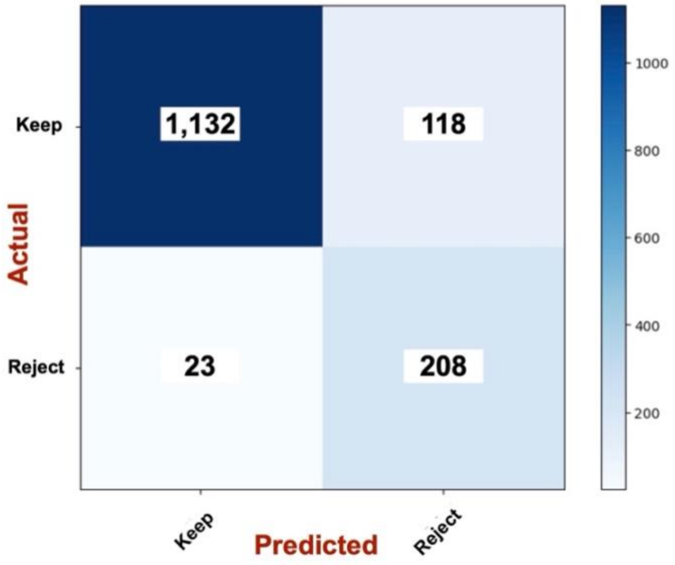

Figure 4. Confusion matrix showing the number of true positives ("keep" correctly predicted as "keep"; n=1,132), true negatives ("reject" correctly predicted as "reject"; n=208), false positives ("reject" incorrectly predicted as "keep"; n=23) and false negatives ("keep" incorrectly predicted as "reject"; n=118) based on the rejection thresholds.

Table 4. Sensitivity, specificity, precision and F1 score based on the rejection thresholds.

Measure	Derivations	Performance
Sensitivity	TP / (TP + FN)	0.9056
Specificity	TN / (FP + TN)	0.9004
Precision	TP / (TP + FP)	0.9801
F1 Score	2TP / (2TP + FP + FN)	0.9414

TP, true positive; FN, false negative; FP, false positive; TN, true negative.

S2: Characteristics of eyes passing and failing quality control

Of the 18,757 eyes excluded after applying the quality control method described above, 3,409 were excluded due to image processing failure (i.e. segmentation failure, typically seen in severely underexposed or overexposed images, or images where regions of interest, such as the optic disc, are hidden due to misalignment). The remaining 15,348 eyes were rejected due to suboptimal processing caused by poor image quality, based on the thresholds in Table 3. There was no significant difference in spherical equivalent refraction or intraocular pressure between the included and excluded eyes (Table 5). Likewise, there was insufficient evidence of an association between ethnic group and the likelihood of being excluded. However, compared to the included participants, those excluded tended to be older (by 2.2 years) and were more likely to be male. There was also statistical evidence of higher mean blood pressure among those excluded, but the difference was clinically negligible (0.4 mmHg).

Table 5. Included vs. excluded participants/eyes. Differences in continuous and categorical variables were tested using the two-sample t-test and chi-squared test of independence, respectively.

Characteristics	Included	Excluded	P
Number of eyes	33,950	18,757	
Age (y)	53.5 ± 8.1	55.7 ± 8.2	<0.001
Male (%)	42.1	44.1	<0.001
Spherical equivalent refraction (D)	-0.47 ± 2.25	-0.44 ± 2.68	0.16
White ethnicity (%)	92.5%	92.1%	0.09
Intraocular pressure (mmHg)	15.6 ± 3.7	15.5 ± 3.9	0.08
Mean blood pressure (mmHg)	97.6 ± 11.2	98.0 ± 11.3	<0.001

References

1. Gibbon S, Muniz-Terrera G, Yii FS, et al. A method for quantifying sectoral optic disc pallor in fundus photographs and its association with peripapillary RNFL thickness. Published online November 13, 2023. doi:10.48550/arXiv.2311.07213

2. UK Biobank - UK Biobank. Published February 9, 2024. Accessed February 21, 2024. https://www.ukbiobank.ac.uk

3. Zhang Z, Yin FS, Liu J, et al. ORIGA(-light): an online retinal fundus image database for glaucoma analysis and research. *Annu Int Conf IEEE Eng Med Biol Soc IEEE Eng Med Biol Soc Annu Int Conf*. 2010;2010:3065-3068. doi:10.1109/IEMBS.2010.5626137

4. Decencière E, Zhang X, Cazuguel G, et al. FEEDBACK ON A PUBLICLY DISTRIBUTED IMAGE DATABASE: THE MESSIDOR DATABASE. *Image Anal Stereol*. 2014;33(3):231-234. doi:10.5566/ias.1155

5. Ritchie CW, Ritchie K. The PREVENT study: a prospective cohort study to identify mid-life biomarkers of late-onset Alzheimer's disease. *BMJ Open*. 2012;2(6):e001893. doi:10.1136/bmjopen-2012-001893

6. cataract dataset. Accessed February 21, 2024. https://www.kaggle.com/datasets/jr2ngb/cataractdataset

7. Porwal P, Pachade S, Kamble R, et al. Indian Diabetic Retinopathy Image Dataset (IDRiD): A Database for Diabetic Retinopathy Screening Research. *Data*. 2018;3(3):25. doi:10.3390/data3030025

8. Taylor AM, Pattie A, Deary IJ. Cohort profile update: The Lothian birth cohorts of 1921 and 1936. *Int J Epidemiol*. 2018;47(4):1042-1060. doi:10.1093/ije/dyy022

9. Kovalyk O, Morales-Sánchez J, Verdú-Monedero R, Sellés-Navarro I, Palazón-Cabanes A, Sancho-Gómez JL. PAPILA: Dataset with fundus images and clinical data of both eyes of the same patient for glaucoma assessment. *Sci Data*. 2022;9(1):291. doi:10.1038/s41597-022-01388-1

10. Bajwa MN, Singh GAP, Neumeier W, Malik MI, Dengel A, Ahmed S. G1020: A Benchmark Retinal Fundus Image Dataset for Computer-Aided Glaucoma Detection. Published online May 28, 2020. doi:10.48550/arXiv.2006.09158

11. Jin K, Huang X, Zhou J, et al. FIVES: A Fundus Image Dataset for Artificial Intelligence based Vessel Segmentation. *Sci Data*. 2022;9(1):475. doi:10.1038/s41597-022-01564-3

12. Ocular Disease Recognition. Accessed February 21, 2024. https://www.kaggle.com/datasets/andrewmvd/ocular-disease-recognition-odir5k

13. Pachade S, Porwal P, Thulkar D, et al. Retinal fundus multi-disease image dataset (Rfmid): A dataset for multi-disease detection research. *Data*. 2021;6(2):1-14. doi:10.3390/data6020014
